# Guanine crystals regulated by chitin-based honeycomb frameworks for tunable structural colors of sapphirinid copepod, *Sapphirina nigromaculata*

**DOI:** 10.1038/s41598-020-59090-4

**Published:** 2020-02-10

**Authors:** Tsubasa Kimura, Mihiro Takasaki, Ryosuke Hatai, Yukiko Nagai, Katsuyuki Uematsu, Yuya Oaki, Minoru Osada, Hiroyuki Tsuda, Takaaki Ishigure, Takashi Toyofuku, Shinji Shimode, Hiroaki Imai

**Affiliations:** 1 School of Integrated Design Engineering, Faculty of Science and Technology, Keio University, 3-14-1 Hiyoshi, Kohoku-ku, Yokohama, 223-8522 Japan; 2 X-star, Japan Agency for Marine-Earth Science and Technology (JAMSTEC), Natsushima-cho 2-15, Yokosuka, 237-0061 Japan; 3 National Museum of Nature and Science, 4-1-1 Amakubo, Tsukuba, 305-0005 Japan; 4 Marine Works Japan Ltd., 3-54-1 Oppama-higashi, Yokosuka, 237-0063 Japan; 5 Institute of Materials and Systems for Sustainability, Nagoya University, Furo-cho, Chikusa-ku, Nagoya, 464-8601 Japan; 6 Tokyo University of Marine Science and Technology (TUMSAT), 4-5-7, Konan Minato-ku, Tokyo, 108-8477 Japan; 7 Manazuru Marine Center for Environmental Research and Education, Graduate School of Environment and Information Sciences, Yokohama National University, 61 Iwa, Manazuru, 259-0202 Japan

**Keywords:** Structural biology, Materials science, Optics and photonics

## Abstract

Sapphirinid copepods, which are marine zooplankton, exhibit tunable structural colors originating from a layered structure of guanine crystal plates. In the present study, the coloring portion of adult male of a sapphirinid copepod, *Sapphirina nigromaculata*, under the dorsal body surface was characterized to clarify the regulation and actuation mechanism of the layered guanine crystals for spectral control. The coloring portions are separated into small domains 70–100 µm wide consisting of an ordered array of stacked hexagonal plates ~1.5 µm wide and ~80 nm thick. We found the presence of chitin-based honeycomb frameworks that are composed of flat compartments regulating the guanine crystal plates. The structural color is deduced to be tuned from blue to achromatic via yellow and purple by changing the interplate distance according to vital observation and optical simulation using a photonic array model. The framework structures are essential for the organization and actuation of the particular photonic arrays for the exhibition of the tunable structural color.

## Introduction

Structural color is generated by a combined effect of diffraction, refraction, reflection, and interference of light due to submicron-scale periodic structures^1–6^. Several organisms have characteristic parts that exhibit specific structural colors. For example, we can observe specific colors on feathers of peacock, necks of pigeon, and wings of morpho butterfly^7–9^. Adult males *Sapphirina nigromaculata*, which are marine zooplanktons, show tunable structural colors originating from a layered structure of guanine hexagonal plates ~1.5 µm wide and cytoplasm under the dorsal body surface^10,11^. A feature of the biological structural color on the Sapphirinid copepod is tunability from achromatic to yellow, red, and blue^12–14^. The iridescence of male sapphirinids has been reported to be optimized to communicate with non-iridescent females having relatively larger twin lens-eyes in the water column^13,14^. The disappearance of the structural color in the males is probably utilized as a defense system against visual predators, such as pelagic fishes^10,11,13,14^. The tunability of the structural colors of planktons is very interesting not only for clarification of the biological function but also for the development of biomimetic display technologies. The variation in the structural color with change in the interplate distance was reported by detailed observation using a cryo-technique for electron microscopy and an optical simulation^5,6,10,11^.

Monoclinic guanine crystals are generally found in the photonic structures of a wide variety of organisms, such as the scales of carp and neon tetras, the eyes of scallops and zebrafish, and the body surfaces of several spiders and chameleons^15–21^. The refractive index of guanine crystals in the direction normal to the (100) plane is 1.83, which is one of the highest values in biological matter^22,23^. Thus, platy guanine crystals exposing wide (100) faces reflect visible light effectively in iridophore cells^24–26^. The coloring portion under the dorsal body surface of adult male sapphirinids consists of arrayed guanine hexagonal plates exposing the (100) faces. A twin structure of three guanine hexagonal plates was characterized by transmission electron microscopy (TEM) with electron diffraction^27–29^. Recently, the presence of an amorphous state was reported to be important for the formation of specifically structured guanine crystals^5,6,27^. However, the regulating mechanism of the guanine crystal arrays has not been studied sufficiently for understanding the essence of the tunable structural colors.

The present article reports the micro- and nanostructures of the coloring portions under the dorsal body surface of the sapphirinid copepod, *S. nigroaculata*. A detailed characterization of micrometer-scale domains exhibiting the structural color clarified the ordered arrays of guanine hexagonal plates that are regulated by flat compartments of a chitin-based honeycomb framework. Moreover, we discuss the spectral tuning mechanism of the frameworks consisting of guanine plates and cytoplasm, on the basis of vital observation and optical simulation of the photonic arrays. Understanding the essence of the organization and regulation of the guanine and chitin-based photonic arrays would shed light on biomimetic engineering for novel coloring and display technologies^24,25,30–32^.

## Results and Discussion

### Macrostructures of coloring portions

**Figure 1:**
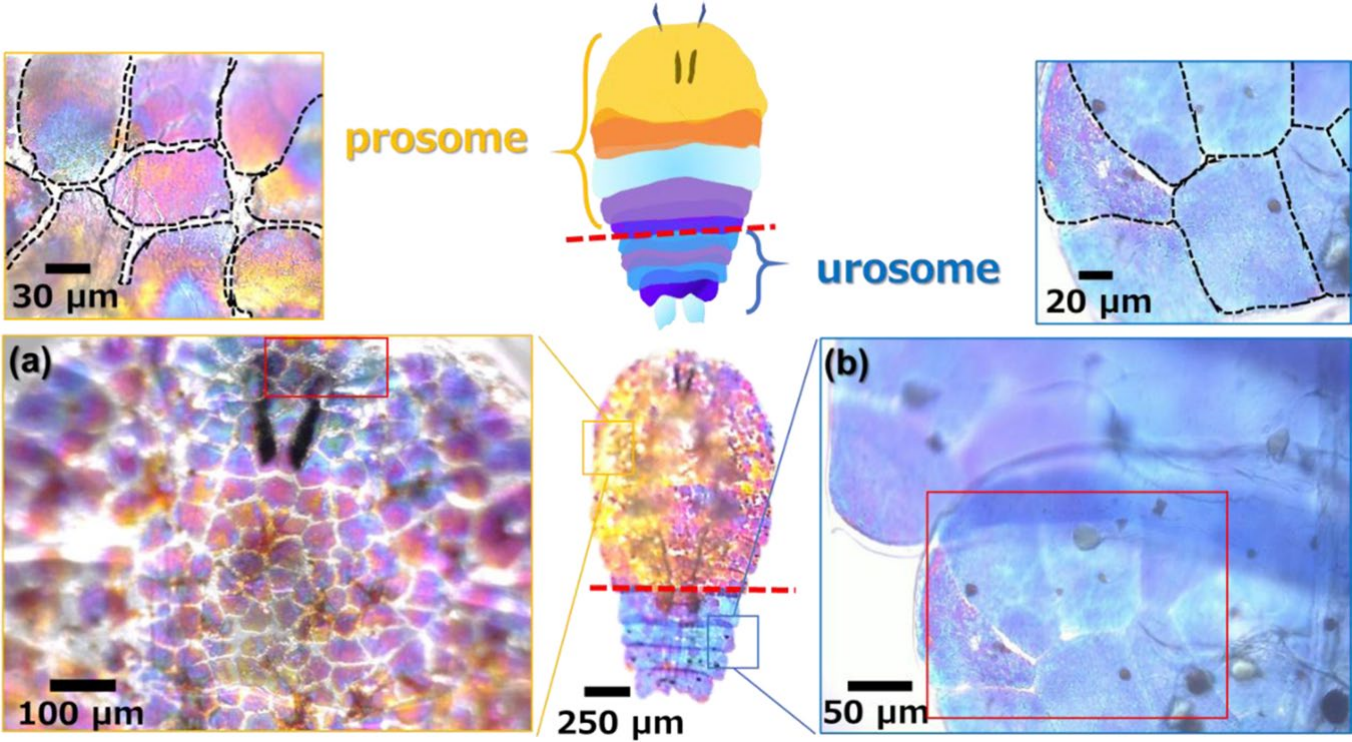
Optical microscope images and schematic illustration of the whole body and the coloring portions of the prosome (**a**) and the urosome (**b**) of *S. nigromaculata*.

The structural color of live *S. nigromaculata* males was monitored by transmitted light under an optical microscope. The males exhibits diurnal-specific color in the morning and is transparent (achromatic) in the afternoon and at night^14^. Figs. 1 and S1 in the ESI show optical microscope images with transmitted light and a schematic illustration of the coloring portions under the dorsal body surface of the prosomal and urosomal parts for a male of *S. nigromaculata*. The structural color of transmitted light varied from blue to achromatic via purple, red, orange, and yellow.

The coloring portions were found to be divided into isolated domains having a similar structural color (Fig. 1a,b). The domains, which ranged from 70 to 100 µm in width, were filled through small gaps in the prosomal and urosomal parts. According to previous studies^10,13,14^, the guanine crystal arrays are stored in an iridophore cell. Moreover, the size of the coloring domains was found to be similar to that of the iridophires in the present study. Thus, we deduced that the isolated domains are contained individually in the cells under the dorsal body surface.

### Honeycomb frameworks regulating hexagonal plates in coloring portions

**Figure 2:**
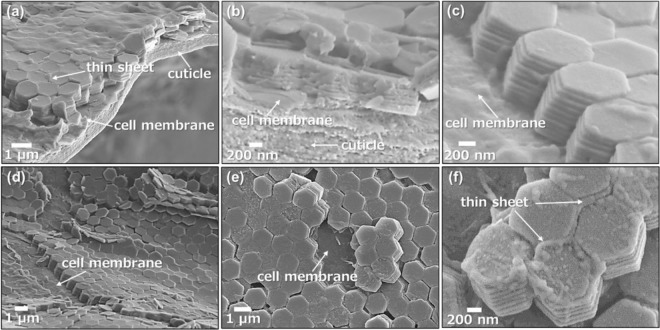
SEM images of the coloring domains under the dorsal body surface of *S. nigromaculata*; bird’s-eye views (**a,c,d**), the cross-sectional view (**b**), and plane views (**e,f**).

We exposed the microstructure of the coloring domains by crushing dried specimens with a tweezer. Scanning electron microscope (SEM) images (Fig. 2) show that the domains comprise ordered arrays of hexagonal units of 1.32 ± 0.15 µm wide (*n*: 50) that are composed of a stacking structure of 8–10 thin plates 86.1 ± 7.6 nm thick (*n*: 50). According to transmission electron microscope images, electron diffraction patterns, and Raman scattering spectra (Fig. S2 in the ESI), the hexagonal plates were assigned to monoclinic β-guanine. As Hirsch *et al*. reported for *S. metallina*^29^, the guanine hexagonal plate is a twin consisting of three crystals exposing a wide (100) plane that are stacked with a rotation of about 60 degrees^29^. The twinned structure was confirmed for the guanine plates in *S. nigromaculata* by the presence of three sets of diffraction spots with rotation angles of ca. 60° (Fig. S2b in the ESI).

**Figure 3:**
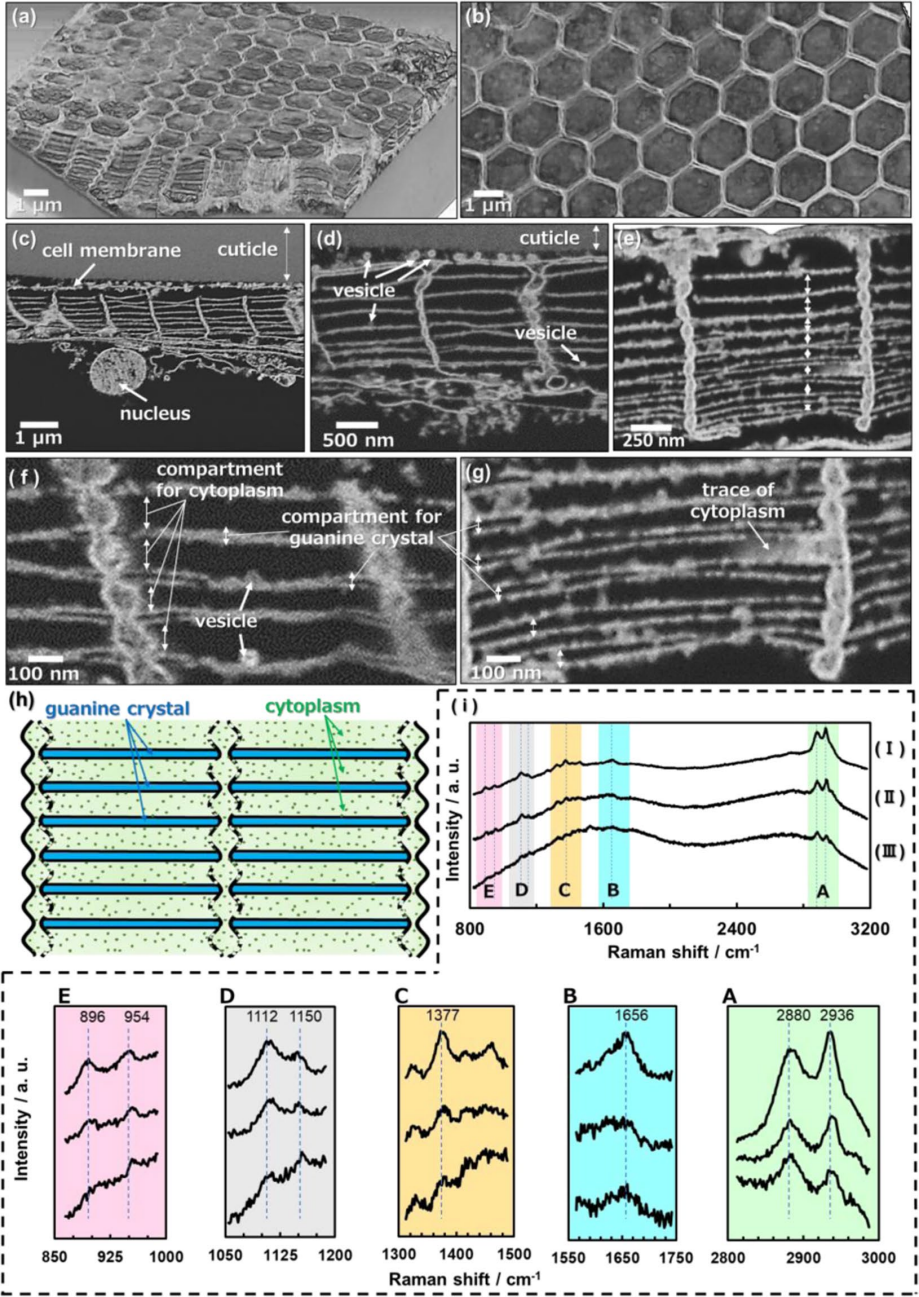
The framework structures in the coloring portions of *S. nigromaculata*. Three-dimensional images reconstructed from serial-sectioning SEM images of consecutive serial sections of a specimen that were collected in an automated manner using FIB-SEM (**a**,**b**); cross-sectional views (**c**–**g)**; schematic illustration of a photonic structure composed of guanine crystal arrays and cytoplasm with the organic framework (**h**); Raman spectra (**i**) for the chitin standard (I) and the dorsal body surface skin (II) and honeycomb framework (III) of *S. nigromaculata*. The assignment of Raman signals (A–E) is shown in Fig. S4 in the ESI.

Thin sheets are observed to partially surround the top and sides of hexagonally packed plates in Fig. 2a,f. However, the whole structure of the frameworks was not recognized in the SEM images. Guanine was then dissolved to clarify the framework structure regulating the crystal plates. The solubility of guanine in water is ca. 0.01 g/dm^3^ (pH 6.0), not zero^33^. Thus, we successfully removed guanine crystals by dissolution in abundant water. Figure 3 shows SEM images for the framework structures in the coloring portions of *S*. *nigromaculata* after removal of the guanine crystals. Honeycomb frameworks are clearly shown in three-dimensional reconstruction images from serial-sectioning SEM images of resin-embedded samples that were collected in an automated manner using a focused ion beam–scanning electron microscope (FIB-SEM) (Fig. 3a,b).

We observed honeycomb frameworks consisting of thin walls ~50 nm thick from top views of the domains (Fig. 3b and S3a–c in the ESI). The honeycomb frameworks are found to be composed of layered compartments consisting of lateral thin sheets ~10 nm thick and vertical walls between the cuticle and the nucleus of an iridophore (Fig. 3c–g). Thick (80–200 nm) and thin (0–50 nm) compartments are alternatively stacked in the honeycomb frameworks (Fig. 3f,g). The vertical walls have a hollow structure consisting of curved skins. According to Raman scattering spectra (Fig. 3i) and energy-dispersive X-ray analysis (Fig. S3d in the ESI), the frameworks are assigned to chitin. The characteristic signals of chitin standard are agreed with those of the samples that were obtained from Sapphirinid copepods. Thus, we identified the dorsal body surface skin and honeycomb frameworks as chitin^34,35^. Here, we successfully observed the chitin-based honeycomb frameworks, including the flat compartments, by a combination of the dissolution of guanine and a freeze-drying technique. The honeycomb-based structural motif is widely utilized in the living world, such as compound eyes of insects and biosilica skeletons in diatoms and glass sponges^36–39^, because the hexagonal cells exhibit a high strength with minimal resources. Thus, the chitin-based frameworks have a reasonable structure for the regulation of the guanine crystal arrays.

The ordered arrays of guanine crystal plates were deduced to be regulated by the chitin-based honeycomb frameworks in the iridophore. Since traces of cytoplasm and vesicles were observed in the thick compartments, it was suggested that the guanine crystals were contained in the thin compartments. The chitin frameworks have not been easily recognized in conventionally dried samples because the compartments shrink with the removal of water in air (Fig. 2). Figure 3h shows a schematic illustration of the organic frameworks that are packed in a photonic structure composed of guanine crystal arrays and cytoplasm.

### Variation of structural color by changing the interplate distance and number of layers

**Figure 4:**
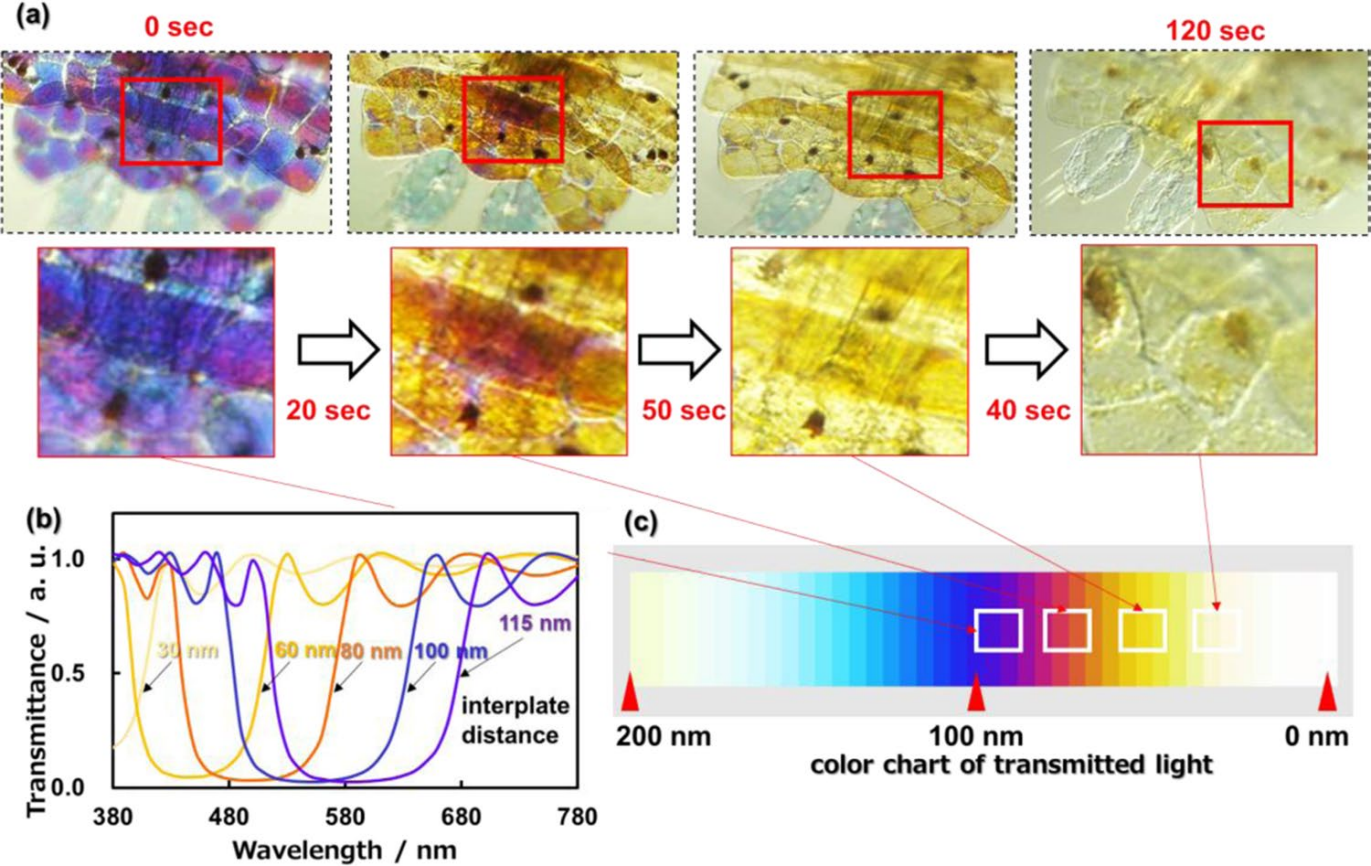
Optical microscope images with the transmitted light of the urosomal part of *S. nigromaculata* (**a**) and simulated spectra (**b**) and color chart (**c**) using the FDTD method; variation of the structural color with the cessation of vital activity by immobilization (**a**); the transmitted spectral variation of transmitted light through an eight-layered structure by cytoplasmic change from 30 to 100 nm (**b**); color chart obtained from simulated spectra with the change of the interplate distance between 0 and 200 nm (**c**). Dark brown dots in the micrographs are pigments^13^.

The coloring part of a living organism was achromatized by immobilization in formalin. Figure 4a shows optical microscope images of the color variation of the urosomal part with the cessation of vital activity by immobilization. The structural color of the transmitted light changed from blue to achromatic via purple, red, and orange in 120 seconds (Fig. S5 in the ESI). The reflecting light of sapphirinid copepods was reported to be tuned by changing the interplate distance between the guanine plates^6,10,12,13^. However, the detailed mechanism for the regulation of the guanine plates has not been clarified in the photonic arrays. The gradual variation of the structural color with immobilization sheds light on the tuning mechanism with changing the interplate distance of the guanine crystals. In the present study, we simulated the structural color by calculating the transmitted light through a layered structure consisting of guanine crystals (*n*: 1.83) and cytoplasm (*n*: 1.30) using the finite-difference time-domain (FDTD) method (Fig. S6 in the ESI). The spectral variation and color chart of transmitted light through an eight-layered structure are shown in Fig. 4b,c by changing the distance of guanine plates from 30 to 100 nm with fixing the plate thickness (80 nm). The change from blue to achromatic via purple and yellow was clearly simulated by decreasing the interplate distance from 100 to 30 nm. The structural color observed with reflected light was monitored with the transmitted spectra from vital observation (Figure S7 in the ESI). The reflection spectra were also successfully simulated by the same procedure.

In previous studies^10,11^, the structural color of the Sapphirinid copepods was suggested to be controlled by changing the distance between the guanine plates. Moreover, the numerical simulation also indicated that the interplate distance is essential for the tunable structural color of the Sapphirinid copepods in the present study. Since the guanine plates are inferred to be stored in the narrow space of the chitin-based frameworks, the thickness of the wide space corresponds the interplate distance (Fig. 3). Thus, we deduce that the structural color is tuned by changing the thickness of the wide spaces in the frameworks. However, the detailed mechanism for the change of the interplate distance regulated by the frameworks has not been clarified sufficiently. Further investigation is required to reveal the actuation system of the tunable structural color.

The change from blue to achromatic via purple and yellow was clearly simulated by decreasing the interplate distance from 100 to 30 nm. Thus, the color variation shown in Fig. 4a and S5 in the ESI is ascribed to shrinkage of the interplate distance with the cessation of vital activity by immobilization. The effect observed after adding formalin to the system is probably due to the high osmotic pressure of the solution, causing water to move from the cells to the surrounding environment.

**Figure 5:**
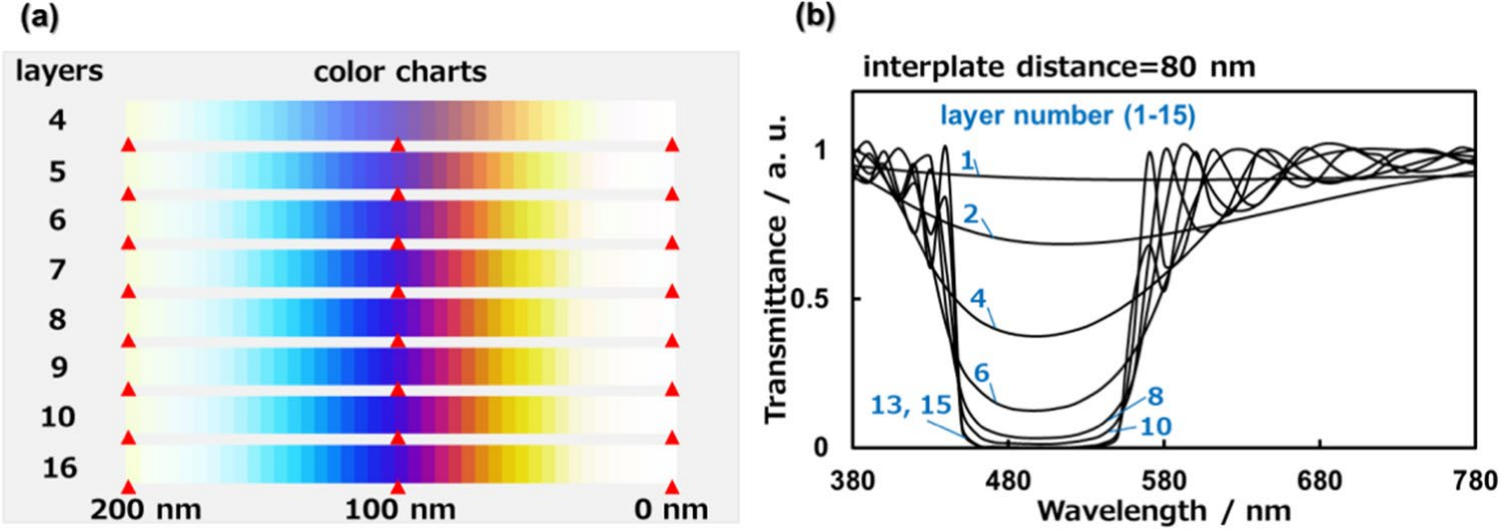
The variation of the structural color with changing the number of layers from 1 to 16 by fixing the plate thickness (80 nm). Simulated color charts of transmitted light with changing the interplate distance from 0 to 200 nm (**a**). Simulated spectra of transmitted light by fixing the plate interplate distance (80 nm) (**b**).

We studied the influence of the number of layers of the guanine plates on the structural color using an optical simulation. The spectral variation and color chart of light transmitted through the layered structure are shown in Fig. 5 by changing the number of layers by fixing the plate thickness (80 nm). The color purity is improved by an increment of the number of layers because the incident light in the wavelength range of 460–560 nm is more efficiently reflected by the multilayered structure (Fig. 5a). However, the simulated spectra are not drastically changed with more than 10 layers (Fig. 5b). Thus, the eight-layered arrays of sapphirinid copepods are deduced to be an ideal structure for the exhibition of vivid tunable colors.

In conclusion, the macro- and microstructures of the coloring portions in sapphirinid copepods, *S. nigroaculata*, were studied by vital observation, detailed characterization with electron microscopy, and optical simulation. The coloring portion comprises domains 70–100 µm wide in iridophores under the dorsal body surface. An ordered array of guanine hexagonal plates ~1.5 µm wide and ~80 nm thick in each domain are regulated by flat compartments in a chitin-based honeycomb framework. Understanding the essence of the organization and regulation of tunable structural colors of *S. nigroaculata* would shed light on biomimetic engineering for novel coloring and display devices.

## Experimental

### Structural analysis of sapphirinid copepods

Plankton samplings were conducted at 35°08.9′N 139°10.5′E in the western part of the Sagami Bay in the south of Japan, on R/V *Tachibana* of the Manazuru Marine Center for Environmental Research and Education, Yokohama National University. Specimens of adult male *S. nigromaculata* were collected by a plankton net (caliber: 80 cm, side length: 3 m, mesh size: 100 μm), and *S. nigromaculata* is a common species that is widely distributed in tropical and subtropical regions^10,13,14^. Culture experiments of *S. nigromaculata* were conducted in a laboratory incubator at 18–20 °C. Living specimens of the males *S. nigromaculata* were sorted from the plankton samples and transferred to a vessel containing filtered seawater.

The living specimens were immersed in deionized water, and water freeze-drying equipment (FD-6500; Kyowa Corporation) was used to obtain freeze-dried samples. (Fig. S7 in the ESI). Cross sections of the dried samples exposed by crushing were observed by SEM and an optical microscope.

The surfaces and cross sections of the male *S. nigromaculata* were coated with osmium for detailed observation using a scanning electron microscope operated (SEM, FEI Helios G4 UX, JEOL JSM-7100) operated at 2.0–15.0 kV. The compositions were identified using Raman scattering spectroscopy and energy-dispersive X-ray analysis (JEOL JED-2300). The micro-Raman was performed using a laser confocal microscope (inVia, Renishaw). The 532 nm excitation laser was focused on the sample surface with a 100× objective lens. The size of the laser spot was about 1 µm in diameter. Chitin standard ((C_8_H_13_O_5_N)_*n*_) was purchased from Kanto Chemical Co. Crystalline parts in the males were characterized by transmission electron microscopy (TEM, FEI Tecnai G2). The samples were dropped with water on a copper grid and crushed with a needle to release crystalline parts from the main body. A suspension containing crystals was quickly dried for a few minutes on a copper grid for TEM observation. Crystalline parts were dissolved to observe the frameworks by immersing specimens in deionized water for several hours. Formalin-fixed samples were prepared in 2.5% glutaraldehyde and 2.0% paraformaldehyde with sea water as a buffer and stained with a heavy metal adhesion operation, with a 2% osmic acid aqueous solution, a 4% uranium acetate aqueous solution, or an aspartic acid Pd solution. The samples were then embedded in Epon 812 (TAAB).

Cross-sectional SEM images showing serial sections of honeycomb frameworks were obtained by cutting the resin-embedded sample every 200 nm using a focused ion beam–scanning electron microscope (FIB-SEM) (FEI Helios G4 UX), and the sample totaled about 8 µm. Three-dimensional structures of honeycomb frameworks were reconstructed by stacking the serial-sectioning SEM images using software (FEI, Amira ver. 6.20).

### Optical simulation

The tunability of the structural colors was discussed based on the simulation of light transmitted through the layered guanine crystal and cytoplasm using a finite-difference time-domain method (FDTD method). Details of the condition were described in the ESI with Figs. S6 and S7.

## Supplementary information

Supplementary information.

## References

[1] Weiner S, Addadi L (1997). Design strategies in mineralized biological materials. J. Mater. Chem..

[2] Dumanli AG, Savin T (2016). Recent advances in the biomimicry of structural colours. Chem. Soc. Rev..

[3] Sun J, Bhushan B, Tong J (2013). Structural coloration in nature. RSC Adv..

[4] Sato K (2016). Optical properties of biosilicas in rice plants. RSC Adv..

[5] Addadi L, Gal A, Faivre D, Scheffel A, Weiner S (2016). Control of Biogenic Nanocrystal Formation in Biomineralization. Isr. J. Chem..

[6] Gur D, Palmer BA, Weiner S, Addadi L (2017). Light Manipulation by Guanine Crystals in Organisms: Biogenic Scatterers, Mirrors, Multilayer Reflectors and Photonic Crystals. Adv. Funct. Mater..

[7] Zi J (2003). Coloration strategies in peacock feathers. Proc. Natl. Acad. Sci. USA.

[8] Yoshioka S, Nakamura E, Kinoshita S (2007). Origin of two-color iridescence in rock dove’s feather. J. Phys. Soc. Japan.

[9] Vukusic P, Noyes J (2003). Photonic structures in biology. Nature.

[10] Gur D (2016). Light-Induced Color Change in the Sapphirinid Copepods: Tunable Photonic Crystals. Adv. Funct. Mater..

[11] Gur D (2015). Structural Basis for the Brilliant Colors of the Sapphirinid Copepods. J. Am. Chem. Soc..

[12] Chae J, Nishida S (1994). Integumental ultrastructure and color patterns in the iridescent copepods of the family Sapphirinidae (Copepoda: Poecilostomatoida). Mar. Biol..

[13] Chae J, Nishida S (1999). Spectral patterns of the iridescence in the males of *Sapphirina* (Copepoda: Poecilostomatoida). J. Mar. Biol. Assoc. United Kingdom.

[14] Takahashi K, Ichikawa T, Tadokoro K (2015). Diel colour changes in male *Sapphirina nigromaculata* (Cyclopoida, Copepoda). J. Plankton Res..

[15] Funt N, Palmer BA, Weiner S, Addadi L (2017). Koi fish-scale iridophore cells orient guanine crystals to maximize light reflection. Chempluschem.

[16] Yoshioka S (2011). Mechanism of variable structural colour in the neon tetra: Quantitative evaluation of the Venetian blind model. J. R. Soc. Interface.

[17] Gur D (2015). The Mechanism of Color Change in the Neon Tetra Fish: A Light-Induced Tunable Photonic Crystal Array. Angew. Chemie - Int. Ed..

[18] Kinkhabwalaa A (2011). A structural and functional ground plan for neurons in the hindbrain of zebrafish. Proc. Natl. Acad. Sci. USA.

[19] Levy-Lior A (2010). Guanine-Based biogenic photonic-crystal arrays in fish and spiders. Adv. Funct. Mater..

[20] Teyssier J, Saenko SV, Van Der Marel D, Milinkovitch MC (2015). Photonic crystals cause active colour change in chameleons. Nat. Commun..

[21] Palmer BA, Gur D, Weiner S, Addadi L, Oron D (2018). The Organic Crystalline Materials of Vision: Structure–Function Considerations from the Nanometer to the Millimeter Scale. Adv. Mater..

[22] Pirie A (1959). Crystals of Riboflavin making up the Tapetum Lucidum in the eye of a Lemur. Nature.

[23] Oaki Y, Kaneko S, Imai H (2012). Morphology and orientation control of guanine crystals: A biogenic architecture and its structure mimetics. J. Mater. Chem..

[24] Iwasaka M, Mizukawa Y, Roberts NW (2016). Magnetic Control of the Light Reflection Anisotropy in a Biogenic Guanine Microcrystal Platelet. Langmuir.

[25] Iwasaka M, Mizukawa Y (2013). Light reflection control in biogenic micro-mirror by diamagnetic orientation. Langmuir.

[26] Levy-Lior A (2008). Biogenic guanine crystals from the skin of fish may be designed to enhance light reflectance. Cryst. Growth Des..

[27] Gur D (2013). Guanine-based photonic crystals in fish scales form from an amorphous precursor. Angew. Chem. Int. Ed..

[28] Palmer BA (2017). The image-forming mirror in the eye of the scallop. Science..

[29] Hirsch A (2017). Biologically Controlled Morphology and Twinning in Guanine. Crystals. Angew. Chemie - Int. Ed..

[30] Choudhury SM (2018). Material platforms for optical metasurfaces. Nanophotonics.

[31] Kashiwagi, H., Asada, H. & Iwasaka, M. Optical behavior of guanine microcrystals from aquatic species upon exposure to a magnetic field. *IEEE Int. Magn. Conf. INTERMAG* 2018 1–5, 10.1109/INTMAG.2018.8508337 (2018).

[32] Mootha A (2019). Refinement of synthetic guanine crystals for fast diamagnetic rotation. AIP Adv..

[33] Hayes, F. N. The influence of solvation of purinic and pyrimidinic bases on the conformational stability of DNA solutions. *Biochim. Biopys.**Acta.* **134**, 204–206 (1967).

[34] Zaja̧c, A., Hanuza, J., Wandas, M. & Dymińska, L. Determination of N-acetylation degree in chitosan using Raman spectroscopy. *Spectrochim. Acta A* **134**, 114–120 (2015). 10.1016/j.saa.2014.06.071 25011040

[35] Ehrlich H (2007). FirstEvidenceofChitinasaComponent of the Skeletal Fibers of Marine Sponges. Part I. Verongidae (Demospongia: Porifera). J. Exp. Zool. B. Mol. Dev. Evol..

[36] Ehrlich, H. *Marine Biological Materials of Invertebrate Origin*. *Springer* (2019).

[37] Wysokowski M, Jesionowski T, Ehrlich H (2018). Biosilica as a source for inspiration in biological materials science. Am. Mineral..

[38] Zhang Q (2015). Bioinspired engineering of honeycomb structure - Using nature to inspire human innovation. Prog. Mater. Sci..

[39] Brunner E (2009). Chitin-based organic networks: An integral part of cell wall biosilica in the diatom thalassiosira pseudonana. Angew. Chemie - Int. Ed..

