# Supplementary material for: Guanine crystals regulated by chitin-based honeycomb frameworks for tunable structural colors of sapphirinid copepod, Sapphirina nigromaculata
Source: Sci Rep. 2020 Feb 10;10:2266. doi: 10.1038/s41598-020-59090-4 (PMC7010661; doi:10.1038/s41598-020-59090-4)
Supplement: Supplementary file 1 — Supplementary information. [file 41598_2020_59090_MOESM1_ESM.docx]

Supplementary Information

**Guanine crystals regulated by chitin-based honeycomb frameworks for tunable structural colors of sapphirinid copepod, *Sapphirina nigromaculata***

Tsubasa Kimura^1^・Mihiro Takasaki^1^・Ryousuke Hatai^1^・Yukiko Nagai^2,3^・Katsuyuki Uematsu^4^・Yuya Oaki^1^・Minoru Osada^5^・Hiroyuki Tsuda^1^・Takaaki Ishigure^1^・Takashi Toyofuku^2,6^・Shinji Shimode^7^・Hiroaki Imai^1※^

**Address**

1: School of Integrated Design Engineering, Faculty of Science and Technology, Keio University, 3-14-1 Hiyoshi, Kohoku-ku, Yokohama, 223-8522, Japan /E-mail^※^: hiroaki@applc.keio.ac.jp

2: X-star, Japan Agency for Marine-Earth Science and Technology (JAMSTEC), Natsushima-cho 2-15, Yokosuka 237-0061, Japan

3: National Museum of Nature and Science, 4-1-1 Amakubo, Tsukuba, 305-0005, Japan

4: Marine Works Japan Ltd., 3-54-1 Oppama-higashi, Yokosuka 237-0063, Japan

5: Institute of Materials and Systems for Sustainability, Nagoya University, Furo-cho, Chikusa-ku, Nagoya 464-8601, Japan

6: Tokyo University of Marine Science and Technology (TUMSAT), 4-5-7, Konan Minato-ku, Tokyo 108-8477, Japan

7: Manazuru Marine Center for Environmental Research and Education, Graduate School of Environment and Information Sciences, Yokohama National University, 61 Iwa, Manazuru, 259-0202, Japan

Corresponding authors

Hiroaki Imai E-mail address: hiroaki@applc.keio.ac.jp

**Figure S1**

**
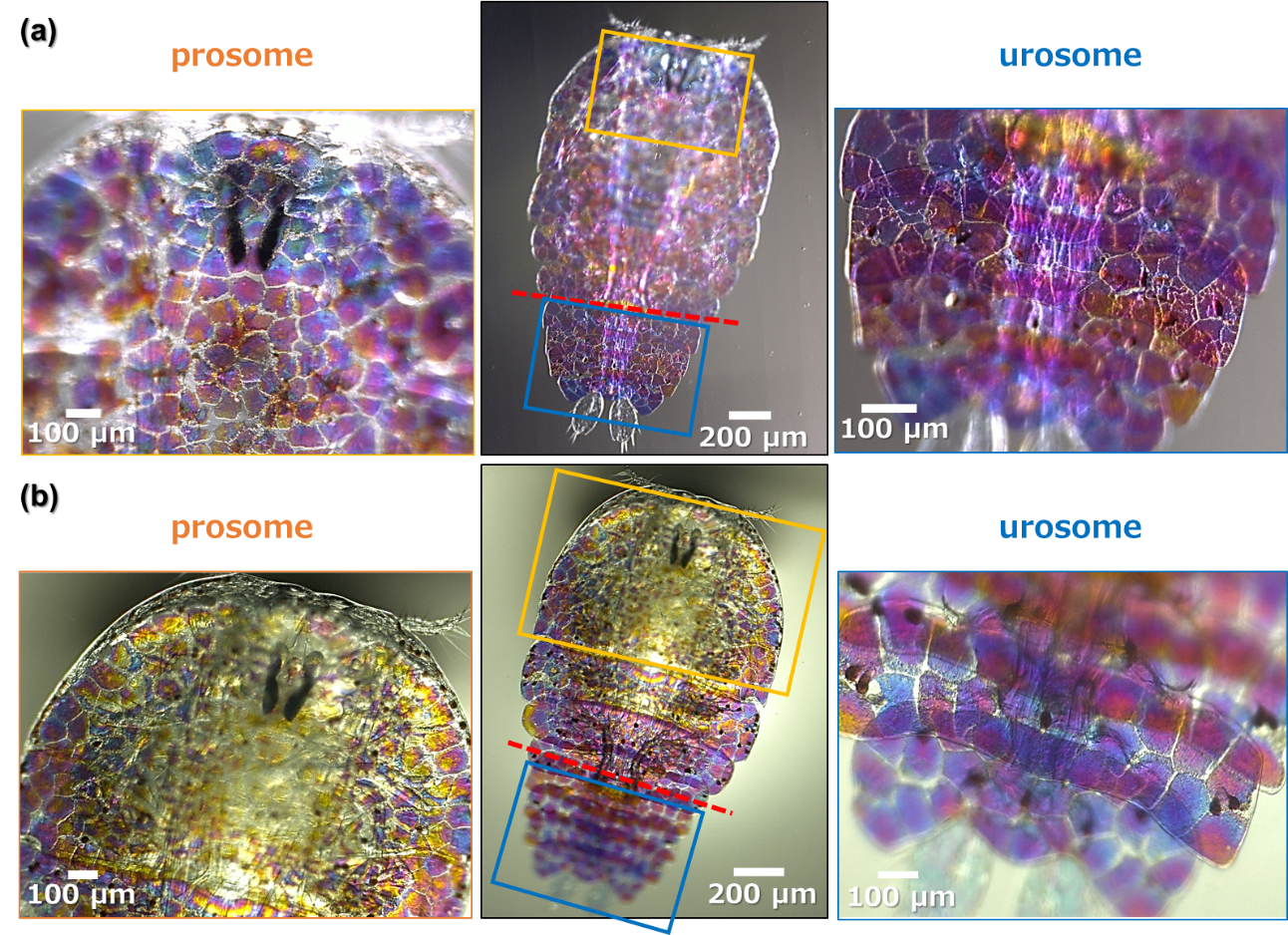
**

Supplementary Figure 1. Optical microscope images of whole bodies and enlarged views of adult male of sapphirinid copepods, *Sapphirina nigromaculata* (a, b)

**Figure S2**

**
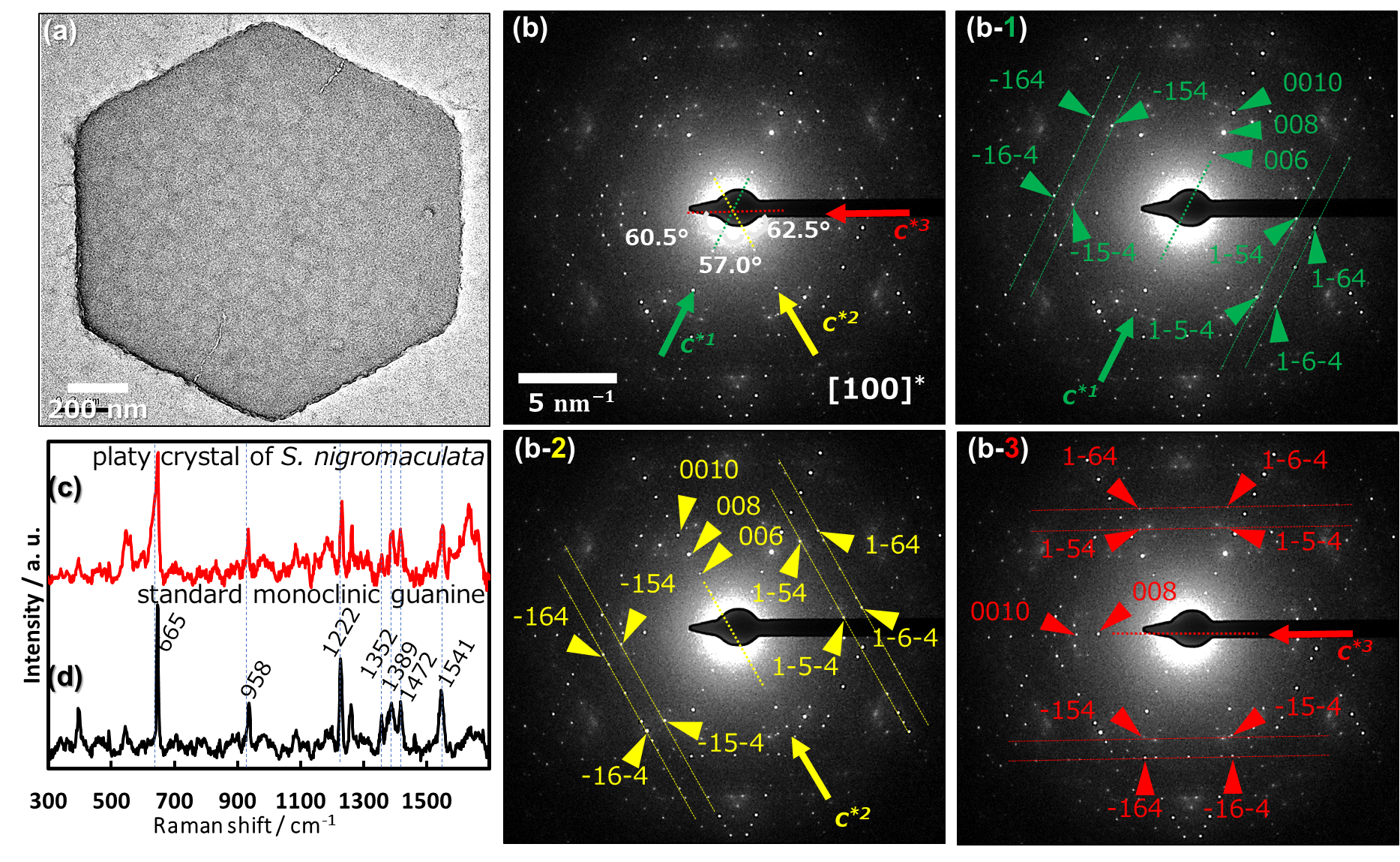
**

Supplementary Figure 2. TEM observation and electron diffraction of guanine plates. A typical TEM image (a) with SAED spots (b) and a typical Raman scattering spectrum (c) for a plate under the dorsal body surface of *S.* *nigromaculata*. We observed three sets of diffraction spots (green, yellow, and red) for monoclinic β-guanine. The center angles of the *c** axes are 57.0°, 60.5°, and 62.5° (*n*: 5). This indicates that the guanine hexagonal plate is a twin consisting of three crystals exposing a wide (100) plane that are stacked with rotation of about 60 degrees. The Raman spectrum is almost the same for that of standard monoclinic guanine crystal (d)^1^.

**Figure S3**


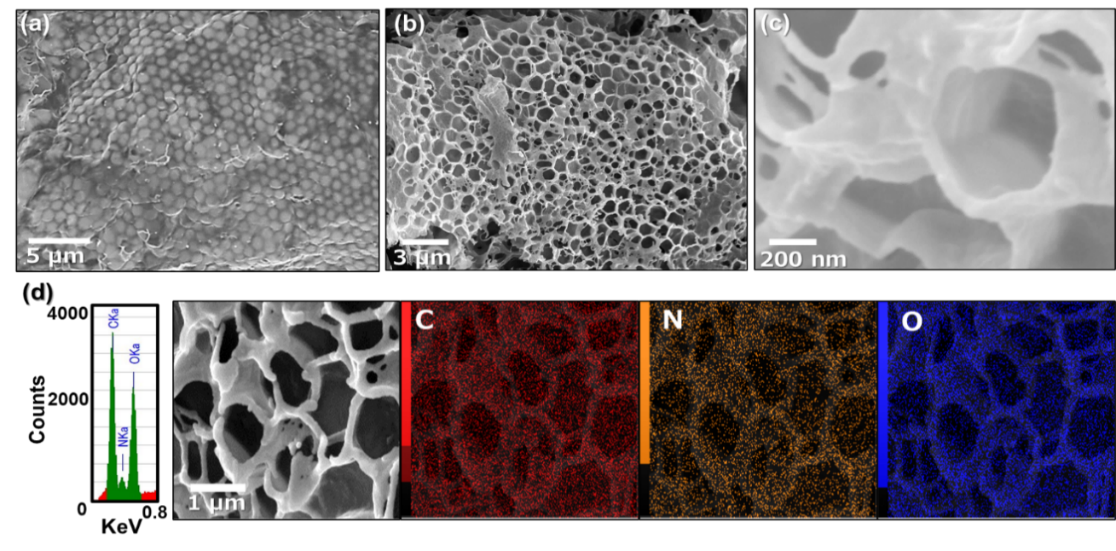


Supplementary Figure 3. SEM and EDX observation of framework structures. SEM and EDX images of the frameworks observed after conventional drying of the specimen of *S.* *nigromaculata*. The organic frameworks (b, c) are observed in the domains. The SEM-EDX image (d) indicates the presence of C, N, and O in the frameworks.

**Figure S4**

**
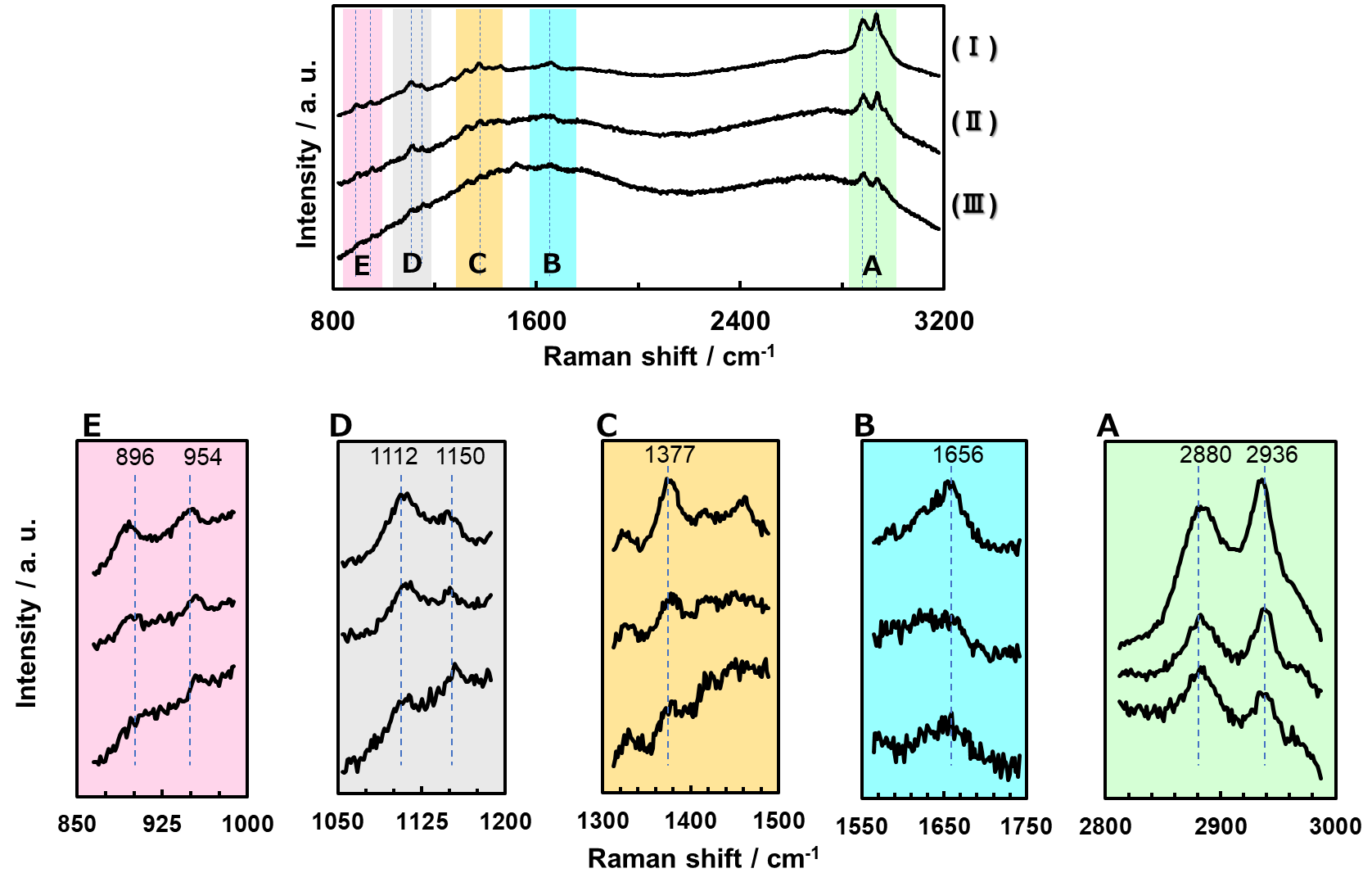
**

Supplementary Figure 4. Raman signals (Figure 3i) are assigned to $v$(CH_3_) (2936 cm^-1^) and $v$(CH_2_) (2880 cm^-1^) in (A), $v$(CO) (1656 cm^-1^) in (B), $\delta$(CH_2_) + $\delta$(CH) + $\delta$(OH) + $v$($\Phi$) (1377 cm^-1^) in (C), $v$(C-O-C) +$v$($\Phi$) +$v$(C-OH) + $v$(C-CH_2_) + $\delta$(CH) (1150 and 1112 cm^-1^) in (D), and $v$(CN) and $v$($\Phi$) (954 and 896 cm^-1^) in (E)^2^.

($\Phi, pyranoid ring; v, stretching; \delta, in$-$plane bending vibrations$).

**Figure S5**


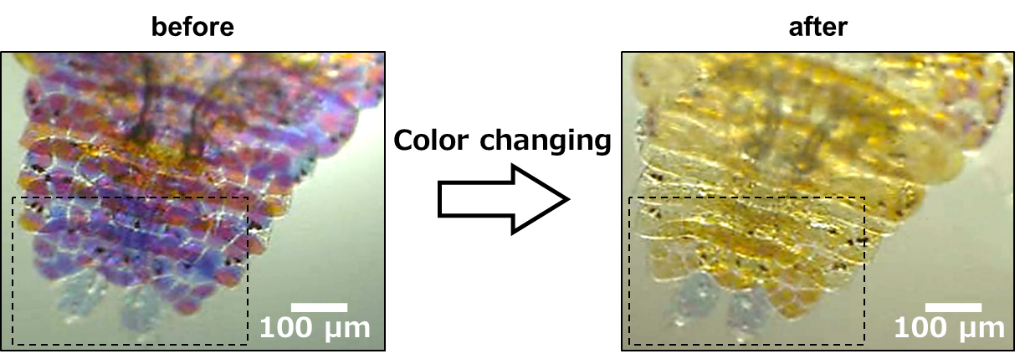


Supplementary Figure 5. Optical microscope images of the structural color change of the specimen of *S.* *nigromaculata* with formalin fixation. The structural color of the transmitted light changed from blue to yellow via purple, red and orange in 120 s.

**Figure S6**


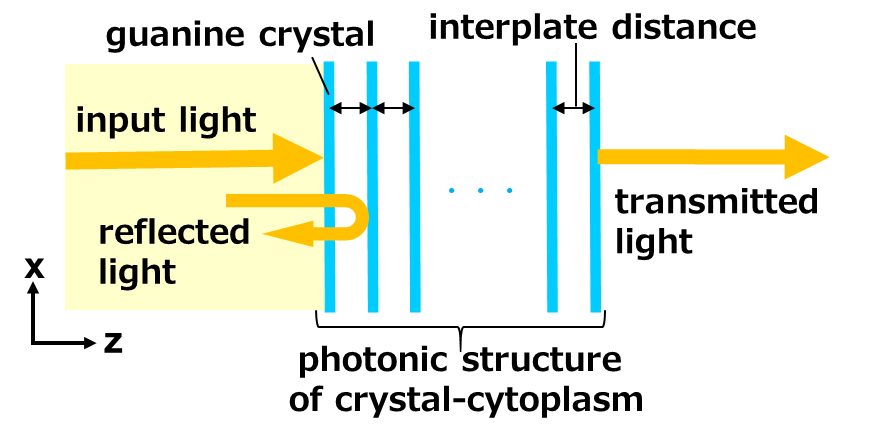


Supplementary Figure 6. Optical simulation using the FDTD method. A schematic model for the finite-difference time-domain (FDTD) method using a Software “RSoft-FullWAVE.” A two-dimension system (the grid size: x =1 nm, z =100 nm) was used for a laminated structure consisting of guanine crystal (*n*: 1.83) and cytoplasm (*n*: 1.30). The thickness of the cytoplasm varied from 20 to 200 nm as a gap of the guanine plates 80 nm thick. The presence of chitin layers is ignored to simplify the simulation.

**Figure S7**


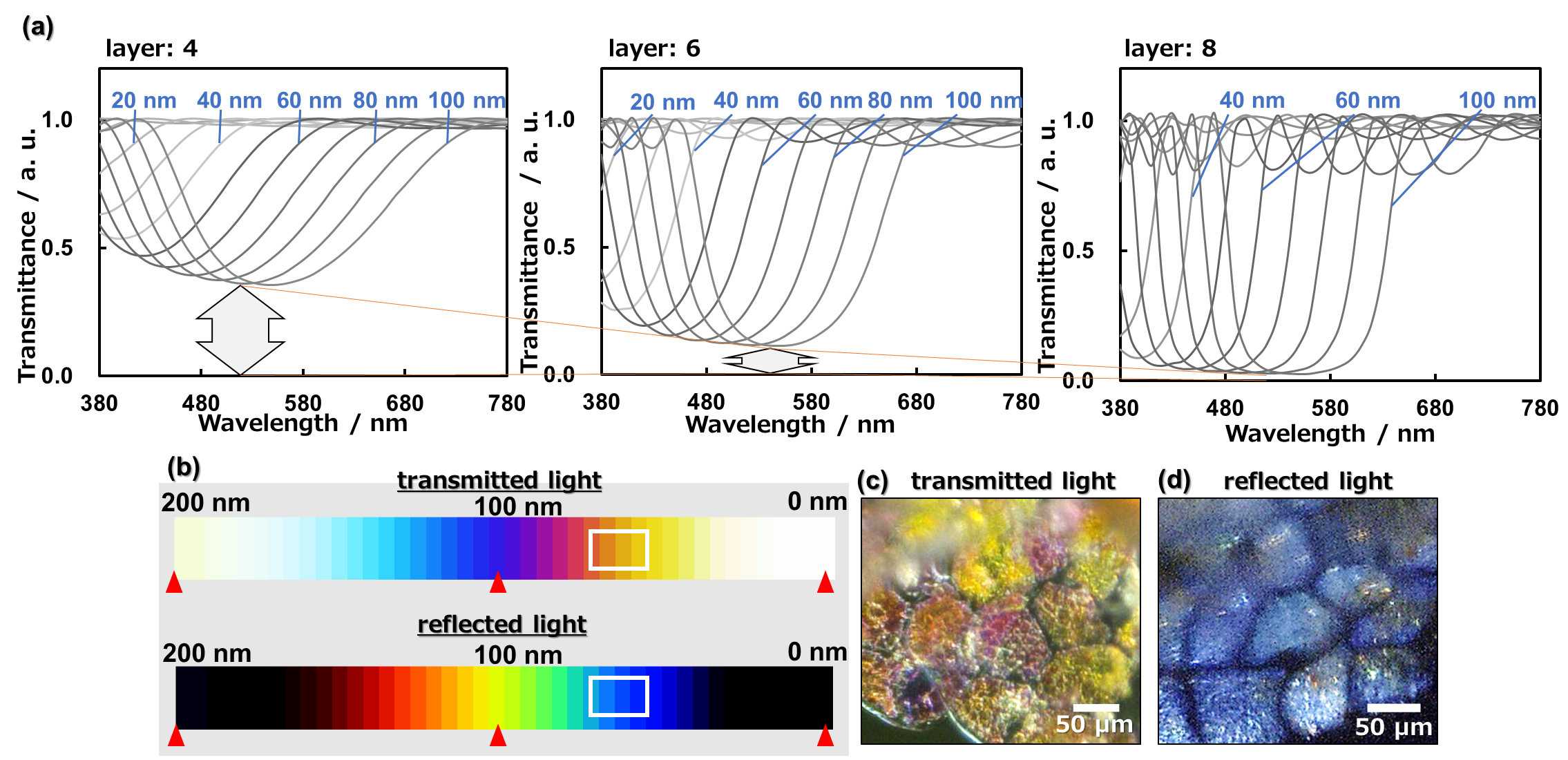


Supplementary Figure 7-1. Simulated transmission spectra with the layer number 4, 6, and 8 in (a). Spectral change in each panel indicates the variation with changing the interplate thickness from 0 to 100 nm. Simulated structural color charts in (b) and optical microscope images of adult male of *S.* *nigromaculata* in (c, d) with transmitted and reflected lights. We simulated the intensity of the transmitted light in the wavelength range of 380-780 nm using a finite-difference time-domain method.

Color charts are quantitatively obtained from the conventional RGB color model by using standard RGB (sRGB) values (Figure S7-2) depending on wavelength (λ) (an international standard established by the International Electrotechnical Commission (1999)). The R, G, and B values are given by the following conversion formulas.

$$R=\sum_{380 nm}^{780 nm} {(sR}_{\lambda}・I_{\lambda})$$

$$G=\sum_{380 nm}^{780 nm} (\mathrm{sG}_{\lambda}・I_{\lambda})$$

$$B=\sum_{380 nm}^{780 nm} (\mathrm{sB}_{\lambda}・I_{\lambda})$$

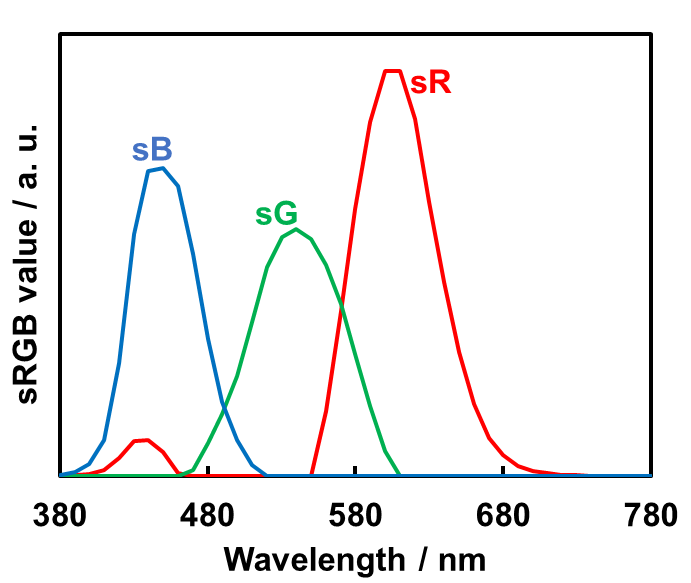


Supplementary Figure 7-2. sRGB values (Chronological Scientific Tables 2018).

**Figure S8**

**
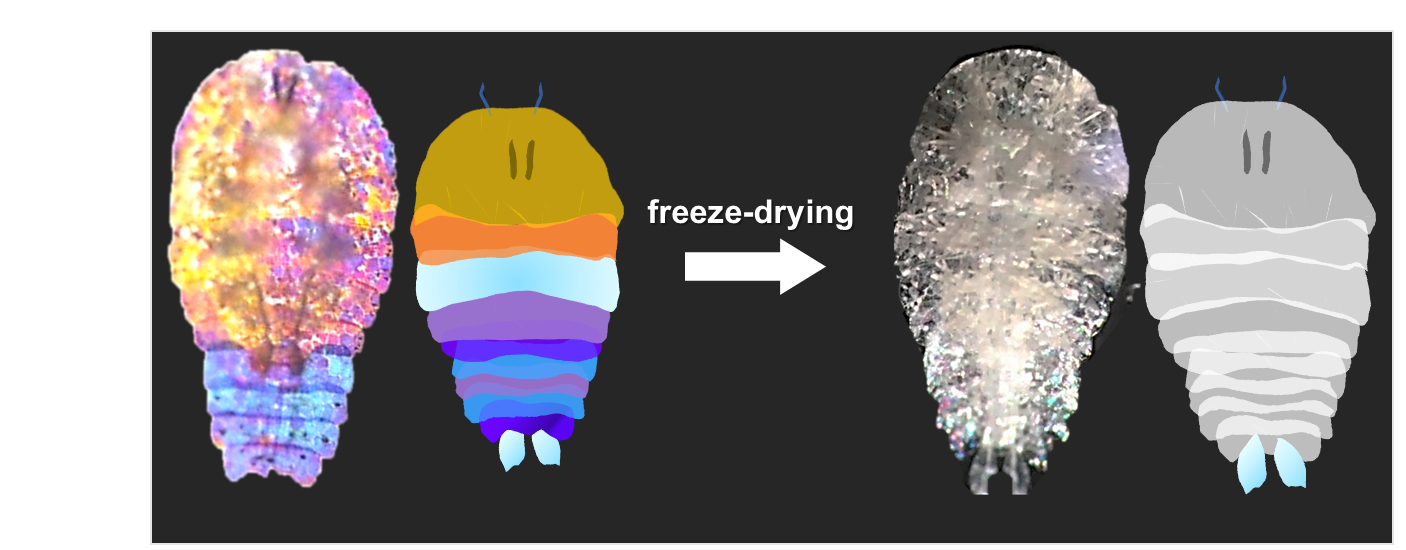
**

Supplementary Figure 8. Change of the structural color by freeze-drying of the specimen of adult male of *S.* *nigromaculata.*

**Reference**

1. Pergolese, B., Muniz-Miranda, M. & Bigotto, A. SERS studies of the adsorption of guanine derivatives on gold colloidal nanoparticles. *Phys. Chem. Chem. Phys.* **7**, 3610–3613 (2005).

2. Zaja̧c, A., Hanuza, J., Wandas, M. & Dymińska, L. Determination of N-acetylation degree in chitosan using Raman spectroscopy. *Spectrochim. Acta Part A Mol. Biomol. Spectrosc.* **134**, 114–120 (2015).
